# Real-world safety and efficacy data of immunotherapy in patients with cancer and autoimmune disease: the experience of the Hellenic Cooperative Oncology Group

**DOI:** 10.1007/s00262-021-02985-6

**Published:** 2021-06-23

**Authors:** Elena Fountzilas, Sofia Lampaki, Georgia-Angeliki Koliou, Anna Koumarianou, Sofia Levva, Anastasios Vagionas, Athina Christopoulou, Athanasios Laloysis, Amanda Psyrri, Ioannis Binas, Giannis Mountzios, Nikolaos Kentepozidis, Athanassios Kotsakis, Emmanouil Saloustros, Anastasios Boutis, Adamantia Nikolaidi, George Fountzilas, Vassilis Georgoulias, Miltiadis Chrysanthidis, Elias Kotteas, Henry Vo, Marinos Tsiatas, Eleni Res, Helena Linardou, Dimitrios Daoussis, Iliada Bompolaki, Anna Andreadou, George Papaxoinis, Dionisios Spyratos, Helen Gogas, Konstantinos N. Syrigos, Dimitrios Bafaloukos

**Affiliations:** 1 Second Department of Medical Oncology, Euromedica General Clinic of Thessaloniki, Gravias 5, 54645 Thessaloniki, Greece; 2 European University Cyprus, Engomi, Cyprus; 3 Pulmonary Department, Lung Cancer Oncology Unit, Aristotle University of Thessaloniki, G. Papanicolaou Hospital, Thessaloniki, Greece; 4 Department of Biostatistics, Hellenic Cooperative Oncology Group, Athens, Greece; 5 Hematology-Oncology Unit, Fourth Department of Internal Medicine, Attikon University Hospital, Medical School, National and Kapodistrian University of Athens, Athens, Greece; 6 Department of Medical Oncology, Bioclinic of Thessaloniki, Thessaloniki, Greece; 7 Department of Medical Oncology, Interbalkan Medical Center, Thessaloniki, Greece; 8 Oncology Department, General Hospital of Kavala, Kavala, Greece; 9 Medical Oncology Unit, S. Andrew Hospital, Patras, Greece; 10 Second Department of Medical Oncology, Hygeia Hospital, Athens, Greece; 11 Section of Medical Oncology, Department of Internal Medicine, Attikon University Hospital, Faculty of Medicine, National and Kapodistrian University of Athens School of Medicine, Athens, Greece; 12 Second Department of Medical Oncology, Metropolitan Hospital, Piraeus, Greece; 13 Fourth Department of Medical Oncology and Clinical Trials Unit, Henry Dunant Hospital Center, Athens, Greece; 14 Department of Medical Oncology, 251 Airforce General Hospital, Athens, Greece; 15 Department of Oncology, School of Health Sciences, University General Hospital of Larissa, University of Thessaly, Larissa, Greece; 16 First Department of Clinical Oncology, Theagenio Hospital, Thessaloniki, Greece; 17 Oncology Department, MITERA Hospital, Athens, Greece; 18 Laboratory of Molecular Oncology, Hellenic Foundation for Cancer Research/Aristotle University of Thessaloniki, Thessaloniki, Greece; 19 Aristotle University of Thessaloniki, Thessaloniki, Greece; 20 German Oncology Center, Limassol, Cyprus; 21 University of Crete, Rethymnon, Greece; 22 First Department of Medical Oncology, Metropolitan Hospital, Piraeus, Greece; 23 Oncology Unit GPP, Sotiria General Hospital, National and Kapodistrian University of Athens School of Medicine, Athens, Greece; 24 Department of Investigational Cancer Therapeutics, University of Texas MD Anderson Cancer Center, Houston, TX USA; 25 Department of Oncology, Athens Medical Center, Athens, Greece; 26 Third Department of Medical Oncology, Agii Anargiri Cancer Hospital, Athens, Greece; 27 Fourth Oncology Department, Metropolitan Hospital, Piraeus, Greece; 28 Department of Internal Medicine, Division of Rheumatology, University of Patras Medical School, Patras University Hospital, Rion, Greece; 29 Oncology Department, General Hospital of Chania, Crete, Greece; 30 Third Department of Medical Oncology, Theagenio Hospital, Thessaloniki, Greece; 31 Second Department of Internal Medicine, Agios Savvas Cancer Hospital, Athens, Greece; 32 First Department of Medicine, Laiko General Hospital, National and Kapodistrian University of Athens School of Medicine, Athens, Greece

**Keywords:** Autoimmune disease, Corticosteroid, Immunomodulatory drugs, Immune-related adverse events, Efficacy, Non-small cell lung cancer

## Abstract

**Background:**

Data on the safety and efficacy of immune checkpoint inhibitors (ICI) in patients with concurrent autoimmune diseases (AID) are limited.

**Methods:**

We performed a retrospective multicenter review of medical records of patients with cancer and underlying AID who received ICI. The primary endpoint was progression-free survival (PFS).

**Results:**

Among 123 patients with pre-existing AID who received ICI, the majority had been diagnosed with non-small cell lung cancer (NSCLC, 68.3%) and melanoma (14.6%). Most patients had a rheumatologic (43.9%), or an endocrine disorder (21.1%). Overall, 74 (60.2%) patients experienced an immune-related adverse event (irAE) after ICI initiation, AID flare (25.2%), or new irAE (35%). Frequent irAEs included thyroiditis, dermatitis and colitis. ICI was permanently discontinued due to unacceptable (8.1%) or fatal (0.8%) toxicity. In patients with NSCLC, corticosteroid treatment at the initiation of immunotherapy was associated with poor PFS (HR = 2.78, 95% CI 1.40–5.50, *p *= 0.003). The occurrence of irAE was associated with increased PFS (HR = 0.48, 95% CI 0.25–0.92, *p *= 0.026). Both parameters maintained their independent prognostic significance.

**Conclusions:**

ICI in patients with cancer and pre-existing AID is associated with manageable toxicity that infrequently requires treatment discontinuation. However, since severe AID flare might occur, expected ICI efficacy and toxicity must be balanced.

**Clinical trial identifier:**

NCT04805099

**Supplementary Information:**

The online version contains supplementary material available at 10.1007/s00262-021-02985-6.

## Introduction

Immunotherapy has led to unprecedented improvement in clinical outcomes compared to standard treatments in selected patients with diverse tumor types [1–3]. Combinations of agents targeting the immune checkpoint receptors cytotoxic T-lymphocyte antigen-4 (CTLA-4), programmed cell death 1 (PD-1), and programmed death-ligand 1 (PD-L1) with chemotherapy, targeted or other immunotherapeutic agents have been approved or are being evaluated in different clinical settings. However, since these receptors play a fundamental role in regulating the immune system, the administration of immunotherapy has been associated with immune-related adverse events (irAE) [4].

Due to this unique toxicity profile, prospective randomized trials evaluating immune checkpoint inhibitors have largely excluded patients with pre-existing autoimmune diseases (AID). Exclusion of these patients was based primarily on concerns about potential increased autoimmune toxicity, requirement for treatment discontinuation and thus compromised efficacy. In addition, patients with AID have amplified immune response, and therefore, treatment with immunotherapy could increase the risk for additional organ inflammation and exacerbated immune-related toxicity. Moreover, patients with AID are often treated with immunomodulatory drugs that have been associated with decreased treatment efficacy and poorer survival [5, 6].

However, in clinical practice, despite lack of robust evidence, patients with well-controlled AID are treated with immune checkpoint inhibitors, especially in cases where clinical benefit is highly expected. Previously published studies have addressed toxicity rates in patients with cancer and underlying AID [7, 8]. Subgroup analysis in 2 prospective clinical trials (SAUL and CheckMate-172) evaluating the safety of atezolizumab and nivolumab, respectively, in patients with AID, demonstrated that the presence of AID did not preclude treatment with immune checkpoint inhibitors [9, 10]. The remaining available data are retrospective, with most of the studies evaluating small numbers of patients [7, 8, 11–18]. Therefore, more robust data on toxicity and efficacy of treatment with immune checkpoint inhibitors in patients with pre-existing AID are needed to ensure their safe use in daily practice.

Real-world data are being increasingly used to evaluate the true effectiveness and safety of innovative therapies. This is especially useful in assessing clinical outcomes and toxicity profiles of drugs in patient populations that are often excluded from randomized clinical trials, such as older patients, patients with comorbidities, poor performance status or patients with AID in case of immunotherapy trials. Two agents (palbociclib for male breast cancer and pembrolizumab for tumors with microsatellite instability) received Food and Drug Administration (FDA)-approvals, partially based on real-world data [19, 20]. Therefore, real-world evidence retrieved from electronic health records, insurance claims, billing databases, registries and patient-generated sources, hold the promise for providing clinically useful information with reduced time, cost, and man-effort.

Our aim was to perform a national, multicenter, retrospective cohort study to evaluate real-world data on safety and efficacy of immunotherapeutic agents in patients with pre-existing AID. We, therefore, performed a retrospective review of medical records of patients with diverse tumor types and underlying AID who received immune checkpoint inhibitors at Departments of Oncology that are affiliated with the Hellenic Cooperative Oncology Group (HeCOG).

## Patients and methods

### Patients

This was a retrospective analysis of patients with diverse tumor types (early stage or metastatic). Patients had received European Medicines Agency (EMA)-approved immune checkpoint inhibitors at HeCOG-affiliated Departments of Oncology from January 2014 to January 2021. Eligible patients were of 18 years or older, with a history of AID, who had received treatment with immune checkpoint inhibitors as any of line of treatment. Pre-existing AIDs were defined according to standard diagnostic criteria; these included but were not limited to rheumatologic, dermatologic, endocrine, neurologic and gastrointestinal inflammatory diseases. Patients were excluded if the AID was diagnosed after initiation of immunotherapy.

Patient clinical data, including demographics, type of AID, treatment data, IRAEs, and outcome data, were obtained from their medical records. Current use of immunosuppressive drugs for the treatment of the underlying AID was also recorded. Immunosuppressive agents were categorized into corticosteroids and other immunomodulatory drugs (cytostatics, antibodies, drugs acting on immunophilins and other) [21]. AID was categorized active and inactive, based on the presence of symptoms or concurrent use of an immunomodulatory drug at the initiation of immune checkpoint inhibitors. Toxicity data [worsening of the AID (flare) and irAE] were retrospectively recorded from the clinicians’ documentations of patient-reported symptoms and laboratory results during scheduled clinical visits or patient hospitalizations. The study was approved by the Institutional Review Board of “G. Papanikolaou” General Hospital (protocol number: 339_4/3/2021).

### Statistical analysis

Descriptive statistics (counts with percentages for categorical and median values with the corresponding ranges for continuous variables) were used to summarize patient characteristics and other variables of interest. The chi-square and the Fisher’s exact test (where more appropriate) were applied to evaluate associations of categorical variables. The primary endpoint of interest was the assessment of progression-free survival (PFS), defined as the time interval from the initiation of immunotherapy to the date of discontinuation (due to any reason), first documented progression, death from any cause or last contact, whichever occurred first. Secondary endpoints included overall survival (OS), best response, presence of irAEs and time to irAE. OS was measured from the day of initiation of immunotherapy to the date of death or last contact. Best response during immune checkpoint inhibitor treatment was defined per physician’s assessment locally at each institution. All irAEs were classified and graded according to the Common Terminology Criteria for Adverse Events (CTCAE, version 4.0). Time to irAE was defined as the time from the initiation of immunotherapy to the development of the irAE. The effect of variables of interest on patients’ outcome in terms of PFS, OS and response rate was examined separately in the entire cohort of patients with available data and among those with advanced non-small cell lung cancer (NSCLC). Cox regression models were applied to estimate the association of variables of interest with progression/mortality rates. Time-dependent covariates were used to evaluate departures from the proportional hazards assumption. The Kaplan–Meier product limit method was applied to assess survival distributions, and the log-rank test was used for comparisons between patient groups. Significance was set at 5%, and all tests were two-sided. Analysis was performed using the SAS v.9.3 (SAS Institute Inc., Cary, NC, USA) statistical software.

## Results

### Patient characteristics

Overall, 123 patients were included in the study, of which 77 (62.6%) were men; median age was 62.1 years of age. The more common cancer diagnosis was lung cancer (*n *= 84, 68.3%), followed by melanoma (*n *= 18, 14.6%) and head and neck cancer (*n *= 6, 4.9%). Treatment with immunotherapy was initiated from January 2014 to January 2021 and was administered as monotherapy in 93 (76.2%) patients. The majority of the patients (*n *= 102, 82.9%) received PD-1 inhibitors as monotherapy. Eight patients (6.5%) were rechallenged with immune checkpoint inhibitors. Detailed patient characteristics are depicted in Table 1.

**Table 1:** Patient and tumor characteristics

	*N* (%)
Age (*N *= 123)	62.1 (35.2,84.5)
*Gender (N *= *123)*	
Female	46 (37.4)
Male	77 (62.6)
*Race (N *= *123)*	
White	123 (100.0)
Other	0 (0.0)
*Serious comorbidities (N *= 122)*	
No	115 (94.3)
Yes	7 (5.7)
*Other comorbidities (N *= *122)*	
No	34 (27.9)
Yes	88 (72.1)
*Active autoimmune disease at initiation of immunotherapy (N *= *123)*	
No	43 (35.0)
Yes	80 (65.0)
*Stage at diagnosis (N *= *122)*	
Early	21 (17.2)
Locally advanced	33 (27.0)
Metastatic	68 (55.7)
*Primary site of cancer (N *= *123)*	
NSCLC	77 (62.6)
Melanoma	18 (14.6)
SCLC	7 (5.7)
Head and neck	6 (4.9)
Urothelial	4 (3.3)
Gastrointestinal	3 (2.4)
Renal	3 (2.4)
Breast	2 (1.6)
Merkel cell	1 (0.81)
Ovarian	1 (0.81)
Sarcoma	1 (0.81)
*PDL-1 expression (N* = *82)*	
Negative	16 (19.5)
Positive	66 (80.5)
*Setting of immunotherapy administration (N* = *123)*	
Adjuvant	9 (7.3)
Locally advanced/metastatic	114 (92.7)
*Line of treatment for metastatic setting (N* = *113)*	
1st line	51 (45.1)
2nd line	53 (46.9)
3rd line and beyond	9 (8.0)
*Immunotherapy agents (N* = *123)*	
CTLA4	4 (3.3)
PD-1/PD-L1	116 (94.3)
Combination CTLA4 and PD-1	3 (2.4)
*Immune checkpoint inhibitor (N* = *122)*	
Pembrolizumab	50 (40.9)
Nivolumab	51 (41.8)
Atezolizumab	8 (6.6)
Durvalumab	5 (4.1)
Ipilimumab	4 (3.3)
Ipilimumab and nivolumab	3 (2.5)
Avelumab	1 (0.8)
*Monotherapy (N* = *122)*	
No	29 (23.8)
Yes	93 (76.2)
*Combined with chemotherapy (N* = *122)*	
No	99 (81.1)
Yes	23 (18.9)

^*^Including long QT syndrome, uncontrolled or significant cardiac disease, (recent myocardial infarction, congestive heart failure, unstable angina and bradyarrhythmias)

*CTLA-4* cytotoxic T-lymphocyte antigen 4, *N* number, *NSCLC* non-small cell lung cancer, *PD-1* programmed cell death protein 1, *PD-L1* programmed death-ligand 1, *SCLC* small cell lung cancer

### Pre-existing autoimmune disease

Among the underlying AID, the majority of patients had a rheumatologic (*n *= 54, 43.9%), or an endocrine disorder (*n *= 26, 21.1%). Patients had been diagnosed with a wide variety of AID, more commonly with proriasis/proriasic arthritis (*n *= 29, 23.6%), rheumatoid arthritis (*n *= 25, 20.3%), and diabetes type I (*n *= 13, 10.6%). Five (4.1%) patients had been diagnosed with two AIDs. Detailed information on pre-existing AIDs is reported in Table 2. At the initiation of immune checkpoint inhibitors, the pre-existing AID was active in 80 (65%) patients. Anti-inflammatory treatment was required in 45 (36.6%) patients at the initiation of immunotherapy; 12 (9.8%) patients received treatment with both corticosteroids and other immunomodulatory medications, while 33 (26.8%) patients received either immunomodulatory agents (excluding corticosteroids) (*n *= 19, 15.4%) or corticosteroids only (*n *= 14, 11.4%). Non-corticosteroid treatments included conventional disease-modifying anti-rheumatic drugs (DMARDs) (cyclosporin, hydroxychloroquine, leflunomide, methotrexate, sulfasalazine, azathioprine), biologic DMARDs (etanercept, ustekinumab) and a phosphodiesterase type 4 (PDE4) inhibitor.

**Table 2:** Type of pre-existing autoimmune disease

Type of pre-existing autoimmune disease	*Ν *(%)
**Rheumatologic**	**54 (43.9)**
Rheumatoid arthritis	25 (20.3)
Sarcoidosis	1 (0.8)
Scleroderma	1 (0.8)
Systemic lupus erythematosus	5 (4.1)
Sjogren	1 (0.8)
Psoriatic arthritis	6 (4.9)
Mixed connective tissue disease	1 (0.8)
Temporal arteritis	1 (0.8)
Polymyalgia rheumatica	3 (2.4)
Vasculitis	7 (5.7)
IgG4 aortitis	1 (0.8)
Arthritis/reiter	2 (1.6)
**Endocrine**	**26 (21.1)**
Hashimoto thyroiditis	12 (9.8)
Graves thyroiditis	1 (0.8)
Diabetes mellitus type I	13 (10.6)
**Gastrointestinal**	**10 (8.1)**
Crohn disease	2 (1.6)
Lymphocytic colitis	1 (0.8)
Ulcerative colitis	6 (4.9)
Primary biliary cholangitis	1 (0.8)
**Dermatologic**	**31 (25.2)**
Psoriasis	23 (18.7)
Vitiligo	7 (5.7)
Lichen planus	1 (0.8)
**Neurologic**	**3 (2.4)**
Myasthenia	2 (1.6)
Myasthenia gravis	1 (0.8)
**Other**	**1 (0.8)**
Pneumonitis	1 (0.8)

*N* number

Bold values indicate statistically significant parameters

### Toxicity

**Fig. 1:**
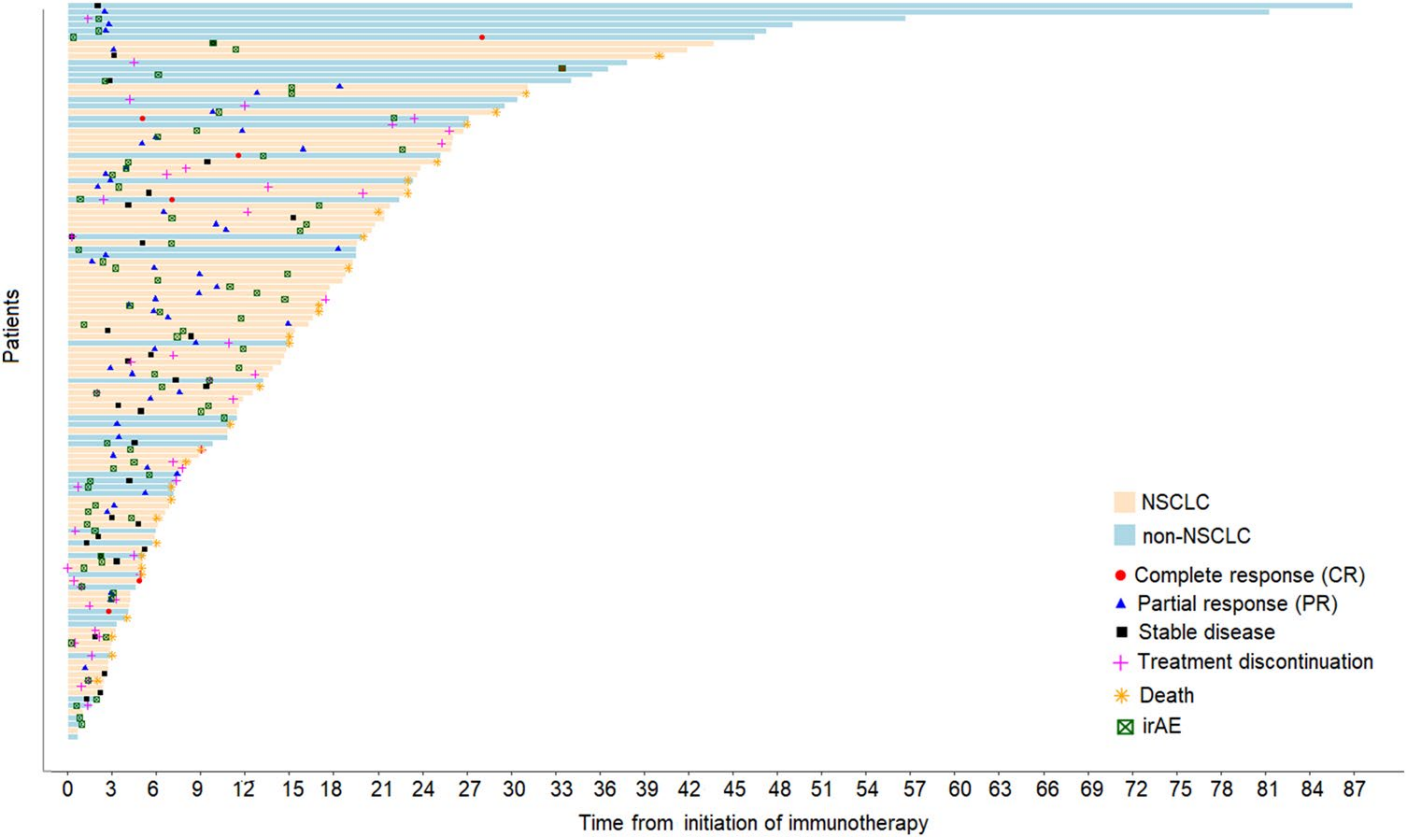
Duration of immunotherapy treatment, toxicity and clinical outcomes of patients treated with immune checkpoint inhibitors

Overall, 74 of 123 patients (60.2%) experienced an irAE after initiation of treatment with an immune checkpoint inhibitor; either exacerbation of the underlying AID (flare) of the pre-existing AID (31, 25.2%), or different irAEs (43, 35%). Ten patients developed both an unrelated irAE and flare of the pre-existing AID. Patients with flare had more commonly underlying dermatologic diseases (12 patients; 38,7%), with the vast majority (10 patients) having been diagnosed with psoriasis. The median time interval between initiation of immunotherapy and development of the irAE was 4.3 [range 0.3–33.4] months (Fig. 1). No association between the occurrence of an irAE and various clinicopathologic factors was identified. In 13 (10.6%) patients, ≥ 2 irAE was reported. Eight patients, all with melanoma, received at least one more treatment with a different immune checkpoint inhibitor (3 with a CTLA-4 inhibitor and 5 with a PD-1 inhibitor). Among those, 6 experienced an irAE during the second line of immunotherapy treatment. Details on irAE are reported in Supplementary Table 1.

The most frequent irAEs experienced by patients on immunotherapy were thyroid dysfunction, skin toxicity and colitis (with/or without endoscopic findings). The presence of irAE did not differ between patients who received anti-PD-1/PD-L1 agents alone or anti CTLA4 (57.1% vs. 60.3% Fisher’s *p *> 0.999). The majority of irAEs were generally mild, while grade 3–4 irAEs were observed in 12 (9.9%) patients. Only in one patient grade 4 exacerbation of psoriasis was reported. There were 2 deaths associated with immunotherapy administration, one was attributed to pneumonitis and one to the flare of the underlying myasthenia. The latter patient was on treatment with corticosteroids and other immunomodulatory agents at the time of immunotherapy initiation for the treatment of advanced NSCLC. He died after one treatment cycle of an anti-PD-1 agent.

Only 22 patients required systemic corticosteroids for management of irAE. The irAE resolved completely in 63 (53.4%) patients. Treatment with an immune checkpoint inhibitor was interrupted in 16 (13.9%) patients, and permanently discontinued due to unacceptable or fatal toxicity in 11 (8.9%) patients. Discontinuation rate (due to any cause) was not found to be associated with the type of agent administered (chi-square *p *= 0.75).

### Efficacy

**Fig. 2:**
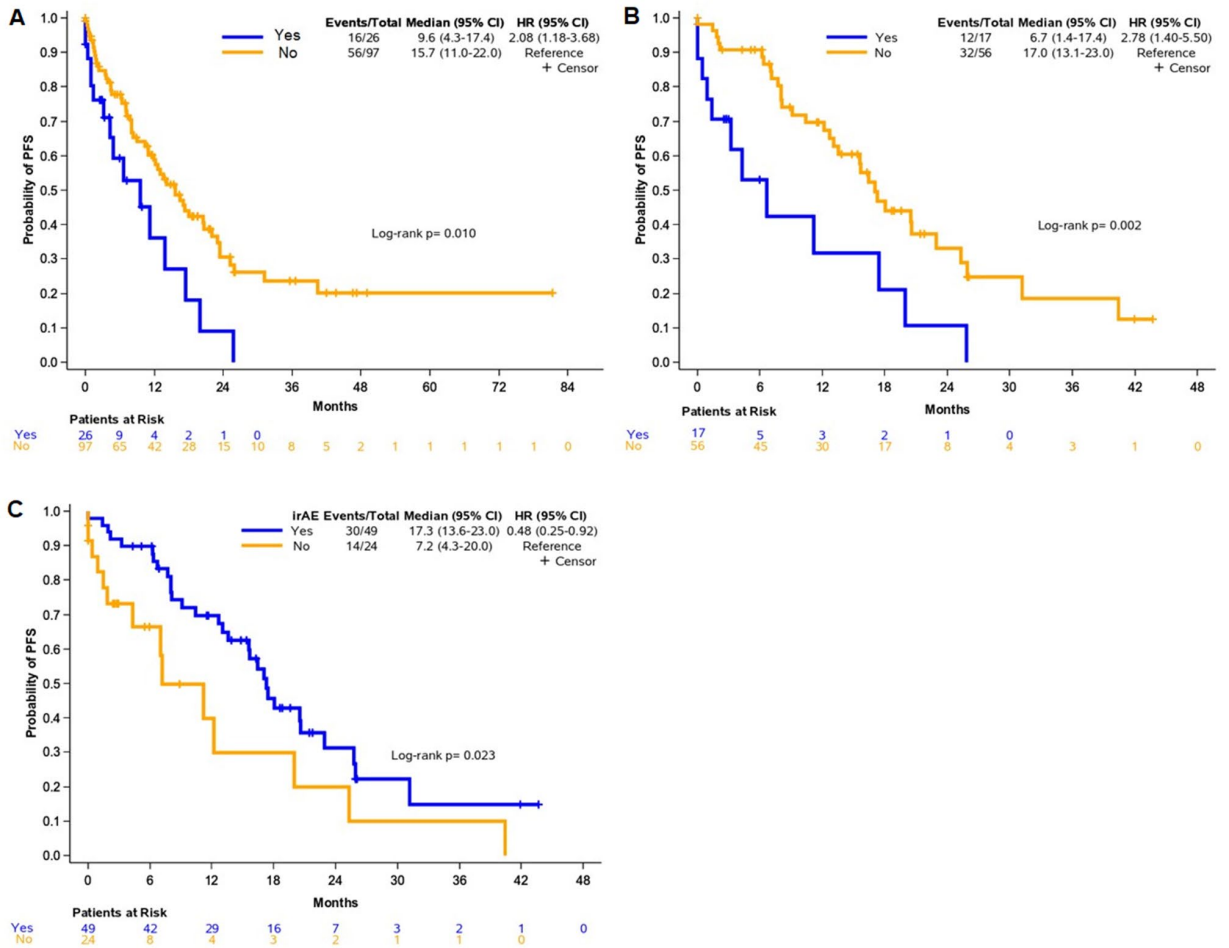
Clinical outcomes. (**A**) Progression-free survival (PFS) based on the use of corticosteroids in the entire cohort, (**B**) PFS based on the use of corticosteroids in patients with advanced non-small cell lung cancer (NSCLC) and (**C**) association of the occurrence of immune-related adverse events (irAEs) with PFS in patients with advanced NSCLC

At the time of analysis, with a median follow-up of 17.4 months (95% CI 13.3–20.6), 30 deaths had occurred. The median OS was 40.5 months (95% CI 27-non-estimable). Objective response to immunotherapy was achieved in 57 of 101 (56.4%) evaluable patients (7 with complete and 50 with partial response), while stable disease was observed in 35 (34.7%) and progressive disease in 9 patients (8.9%) (Fig. 1). We found no association between the use of immunomodulatory treatment (corticosteroids and/or steroid sparing agents) at the time of immune checkpoint inhibitor initiation and response to immune checkpoint inhibitor treatment (*p *= 0.44). We also found no association between the development of AID flare and response to immunotherapy (*p *= 0.73). We did, however, observed an association between the use of corticosteroids as treatment for the AID at the time of initiation of immune checkpoint inhibitors with shorter PFS (HR = 2.08, 95% CI 1.18–3.68, Wald’s *p *= 0.012) (Fig. 2A). There was no association of immunomodulatory agent use (excluding corticosteroids) at immunotherapy initiation and PFS (*p *= 0.22).

Analysis was performed separately in patients with advanced NSCLC, who represented the largest proportion of our patient population. The median OS and PFS for patients with advanced NSCLC, regardless of line of treatment, were 29.1 (95% CI 22.7–30.7) and 16.5 (95% CI 11.2–20.0) months, respectively. Similarly, patients with NSCLC who were on corticosteroid treatment at the initiation of immunotherapy had poorer PFS compared to those who did not (HR = 2.78, 95% CI 1.40–5.50, *p *= 0.003) (Fig. 2B). In addition, the occurrence of an irAE was associated with increased PFS in this patient population (HR = 0.48, 95% CI 0.25–0.92, *p *= 0.026) (Fig. 2C). Detailed data on univariate analysis in the total population and in patients with advanced NSCLC are shown in Table 3.

**Table 3:** Cox univariate regression for parameters of interest with respect to PFS and OS in the entire cohort and among patients with advanced non-small cell lung cancer

	*OS*			*PFS*		
	Event/Total	HR (95% CI)	*p* value	Event/Total	HR (95% CI)	*p* value
Entire cohort						
Immunomodulatory drug use (excluding corticosteroids)						
No	24/92	Reference	–	55/92	Reference	–
Yes	6/31	0.79 (0.32–1.95)	0.616	17/31	1.06 (0.61–1.83)	0.844
Corticosteroid use						
No	25/97	Reference	**–**	56/97	Reference	**–**
Yes	5/26	1.72 (0.64–4.62)	0.279	16/26	2.08 (1.18–3.68)	**0.012**
irAE						
No	14/49	Reference	–	28/49	Reference	–
Yes	16/74	0.55 (0.27–1.12)	0.099	44/74	0.63 (0.39–1.02)	0.059

	*OS*			*PFS*		
	Event/Total	HR (95% CI)	*p* value	Event/Total	HR (95% CI)	*p* value
Advanced NSCLC						
Immunomodulatory drug use (excluding corticosteroids)						
No	14/55	Reference	–	32/55	Reference	–
Yes	4/18	1.04 (0.34–3.18)	0.948	12/18	1.73 (0.88–3.38)	0.111
Corticosteroids use						
No	14/56	Reference	–	32/56	Reference	–
Yes	4/17	2.11 (0.67–6.71)	0.204	12/17	2.78 (1.40–5.50)	**0.003**
irAE						
No	5/24	Reference	–	14/24	Reference	–
Yes	13/49	0.65 (0.23–1.86)	0.425	30/49	0.48 (0.25–0.92)	**0.026**

*CI* confidence interval, *HR* hazard ratio, *irAE* immune-related adverse event, *NSCLC* non-small cell lung cancer, *PFS* progression-free survival, *OS* overall survival

Bold values indicate statistically significant parameters

Multivariate modeling adjusting for PD-L1 expression (which was univariately associated with PFS) was applied to estimate the independent effect of corticosteroid use and irAE occurrence on PFS in patients with advanced NSCLC. The use of corticosteroid treatment for AID and the development of irAE maintained their independence prognostic significance for PFS in multivariate analysis (HR = 2,32, 95% CI 1.04–5.18, *p *= 0.040 and HR = 0.37, 95% CI 0.16–0.88, *p *= 0.024, respectively), while negative PD-L1 expression lost its unfavorable prognostic value (HR = 3.01, 95% CI 0.90–10.05, *p *= 0.073).

## Discussion

**Fig. 3:**
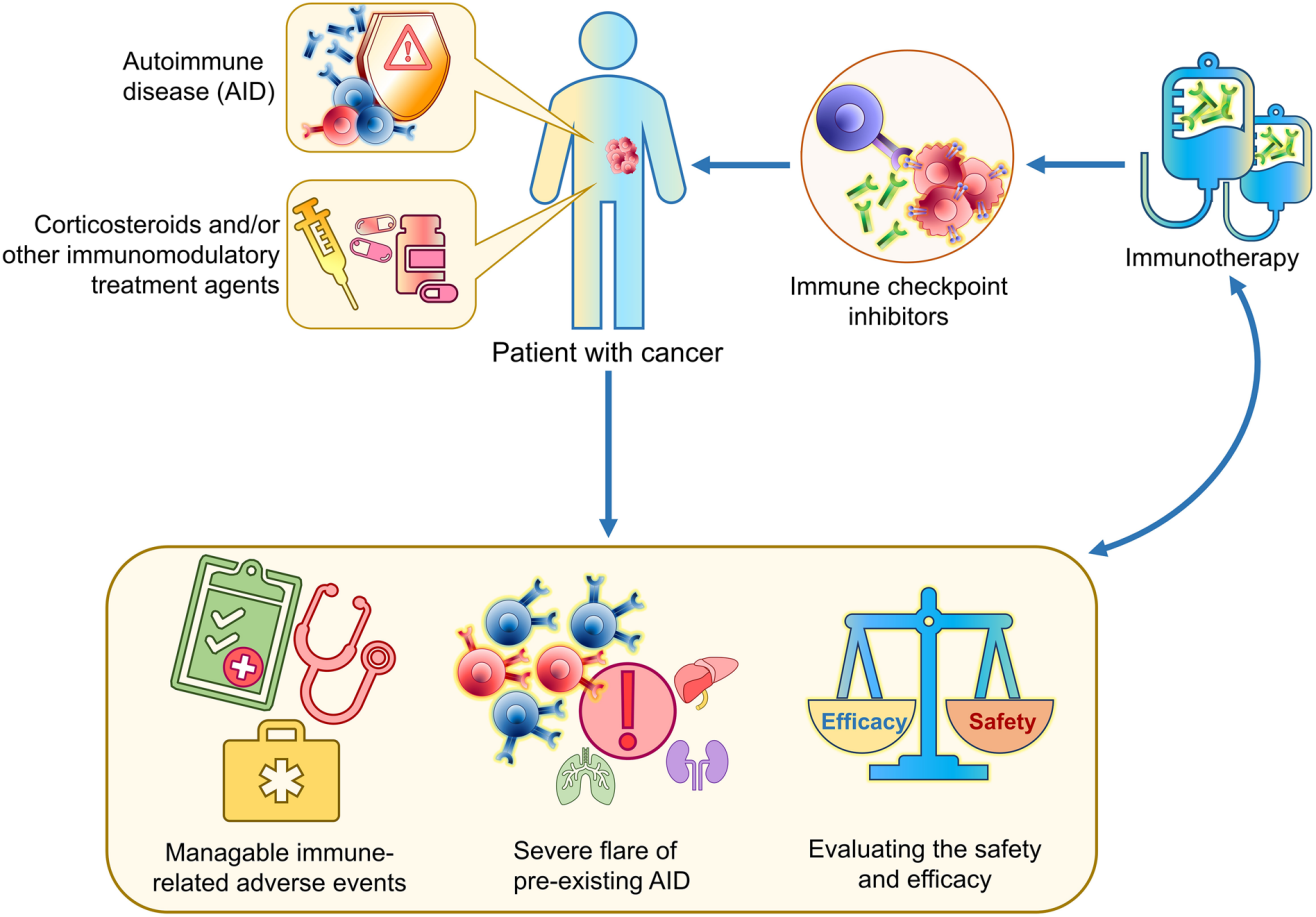
Immunotherapy for treatment of patients with cancer and underlying autoimmune disease (AID). The administration of immunotherapy to patients of our study was associated with manageable adverse events that infrequently required permanent discontinuation of treatment. However, since severe flare of the underlying AID might occur in selected patients, expected efficacy and possible toxicity need to be balanced before treatment administration

This is the largest multicenter study to date, to the best of our knowledge, evaluating the safety and efficacy of immune checkpoint inhibitors in patients with cancer and pre-existing AID. The administration of immunotherapy to patients of our study was associated with manageable adverse events that infrequently required permanent discontinuation of treatment (Fig. 3). However, severe flare of the underlying AID occurred in selected patients, thus underlying the importance of balancing expected efficacy and possible toxicity in these patients. Outcome analysis was performed in all patients, but also separately in patients with NSCLC, representing a more homogenous group. We observed that corticosteroid therapy at initiation of immunotherapy was associated with shorter PFS in patients with NSCLC, which was also confirmed in multivariate analysis. Finally, the occurrence of an irAE in patients with NSCLC receiving immune checkpoint inhibitors was independently associated with longer PFS.

In our study, an exacerbation of the underlying AID was reported in 25% of patients, while an additional 35% developed different irAEs. Most of these adverse events were mild and led to treatment discontinuation in only 8% of patients. Previous studies have similarly reported low discontinuation rates, ranging from 0 to 18% [9, 13, 14, 18, 22–24]. Higher discontinuation rates have been also described, however, in those studies, small number of patients was included, and therefore, these data need to be addressed with caution [17, 25]. Treatment-related deaths have rarely been reported in retrospective studies evaluating immunotherapy in patients with AID and cancer [11, 13, 14]. In one study, there was only one death possibly related to immunotoxicity [18]. In our study, we recorded two immunotherapy-associated deaths, attributed to pneumonitis and flare of the underlying myasthenia, respectively. On the contrary, a significant number of patients did not develop either an exacerbation of the pre-existing AID or different irAEs. Therefore, additional factors seem to influence autoimmunity mechanisms in patients receiving immunotherapy.

Similarly to previous studies, our case series included patients with a wide variety of AID, thus complicating drawing conclusions about specific disorders [12–16, 18, 22]. Only two studies focus on specific AID, inflammatory bower disease [11] and rheumatoid arthritis [24], respectively. The small number of patients with AID that receive treatment with immunotherapy in clinical practice limits analysis on specific AID. Since there are no data on whether all AIDs are associated with similar risk of disease flare or development of other irAEs after treatment with immunotherapy, generalizations need to be made with caution.

Several differences in our patient population, including diverse tumor types and disease stage might have compromised outcome analysis. Therefore, we focused our outcome analysis on a more homogenous group, comprising patients with advanced NSCLC. Importantly, the use of corticosteroids, but not other immunomodulatory agents, at the initiation of immunotherapy treatment was associated with shorter PFS, in the total patient population and in patients with NSCLC. Information on the use of immunosuppressive drugs was relevant to the treatment of the pre-existing AID. However, information on the administration of corticosteroids for other medical purposes, which might have affected patient outcomes [26], was not available. Other investigators also observed that patients who were on immunomodulatory drugs when they received immunotherapy had shorter PFS [18] or lower response rates [14]. Based on this data and taking into account retrospective and case report evidence suggesting that the administration of certain immunomodulatory drugs may not negatively impact clinical outcomes, investigators have suggested strategies to avoid the use of corticosteroids or to replace them with other agents that do not seem to interfere with the efficacy of immunotherapy [27]. To address safety and efficacy concerns, ongoing clinical trials are evaluating the use of immune checkpoint inhibitors in patients with AID and diverse tumor types (i.e., NCT03816345).

Limitations of our study include its retrospective nature, the lack of equal representation of different tumor types and the wide range of AIDs. Strengths of our study include the large patient population, the participation of multiple centers across the country, the inclusion of both efficacy and toxicity detailed data and, finally, the long follow-up for the majority of the patients.

As the number of patients who will receive immunotherapy as part of their cancer treatment significantly increases, it is critical to ensure robust efficacy and safety data for the use of immune checkpoint inhibitors and their combinations in patients with underlying AID. In clinical practice, several factors need to be taken into consideration, including the predicted benefit depending on published data based on tumor type and/or tumor molecular and immune profile, risk of toxicity, type of AID, availability of alternative treatments and patient preference. To date, no consensus guidelines have been proposed to facilitate decision making for these critical clinical issues [27]. Investigators have proposed personalized treatment approaches to address toxicity issues in patients with pre-existing AID who need to receive immunotherapy [27]. Additionally, it is important to expand our understanding on pathogenicity of autoimmunity and identify predictive biomarkers for toxicity to accurately identify patients who will benefit from immune checkpoint inhibitors, while sparing the rest from unnecessary toxicity. Registry and real-world data studies will enable the accumulation of comprehensive data and shed some light into safety and efficacy issues in this vulnerable patient population.

In conclusion, the administration of immune checkpoint inhibitors in patients with cancer and pre-existing AID leads to manageable irAE that infrequently require permanent discontinuation of immunotherapy. However, since severe flare of the pre-existing AID might occur, expected efficacy must be balanced against potential toxicity issues, before initiation of immune checkpoint inhibitors. Importantly, immunotherapy needs to be administered with caution to patients who are under corticosteroid treatment, since certain immunomodulatory drugs might negatively impact their prognosis. Future studies need to prospectively evaluate the administration of immune checkpoint inhibitors and their combinations to large numbers of patients with a wide range of AID to ensure the efficacy and safety of these agents, also in relation to corticosteroids.

### Supplementary Information

Below is the link to the electronic supplementary material.

Supplementary file1 (XLSX 11 kb)

## Data Availability

All data are available upon reasonable request.
